# Molecular surveillance based on anaplasmosis in domestic small ruminants: First report on zoonotic *Anaplasma capra* and phylogenetic insights from Faisalabad, Pakistan

**DOI:** 10.1371/journal.pone.0305412

**Published:** 2024-09-06

**Authors:** Muhammad A. Razzaq, Muhammad Imran, Farhan Ahmad Atif, Rao Z. Abbas, Mughees A. Alvi, Ayman A. Swelum, Zia-ud-Din Sindhu, Muhammad K. Khan, Muhammad A. Sabir Mughal, Adil Khan, Wen-Feng Wu

**Affiliations:** 1 Department of Parasitology, University of Agriculture, Faisalabad, Pakistan; 2 Medicine Section, Department of Clinical Sciences, College of Veterinary and Animal Sciences, Jhang, Sub-Campus University of Veterinary and Animal Sciences, Lahore, Pakistan; 3 Department of Clinical Medicine and Surgery, University of Agriculture, Faisalabad, Pakistan; 4 Department of Animal Production, College of Food and Agriculture Sciences, King Saud University, Riyadh, Saudi Arabia; 5 Department of Botany and Zoology, Bacha Khan University Charsadda, Charsadda, Khyber Pakhtunkhwa, Pakistan; 6 Department of Biology, Mount Allison University, Sackville, New Brunswick, Canada; 7 Department of Radiology, Ditmanson Medical Foundation Chia-Yi Christian Hospital, Chiayi, Taiwan; Kerman University of Medical Sciences, ISLAMIC REPUBLIC OF IRAN

## Abstract

*Anaplasma* is an intracellular alphaproteobacteria that infects diverse blood cell types in animal hosts including small ruminants. Epidemiological and risk factors information on zoonotic anaplasmosis with respect to anaplasmosis in sheep and goats are scarce. Therefore, the objective of the current study was to estimate the prevalence, risk factors of anaplasmosis and phylogenetic investigation of *A*. *capra* in sheep and goats from Faisalabad district, Pakistan. Briefly, 384 blood samples were randomly collected from sheep and goats of Faisalabad district, Pakistan, during January to May 2022. The samples were processed for the detection of *Anaplasma* targeting *16S rRNA* gene using PCR. The data regarding disease determinants were collected using a predesigned questionnaire. Out of 384 samples, 131 samples were found positive for *Anaplasma* spp. with a prevalence rate of 34.11%. The results indicated a significantly higher prevalence of anaplasmosis in goats (41.88%) compared to sheep (22.00%). In addition, the chi square indicated that housing type, tick infestation, gender, tick control practices, age, mix farming, and hygiene were significantly associated with the occurrence of disease. The analysis of multivariate logistic regression expressed gender as the significant risk factor (*p* = 0.0001, OR = 1.757, CI = 1.305–2.366). The acquired sequences revealed four novel isolates of *A*. *capra* (Genbank accession numbers ON834323, ON838209, ON838210, and ON838211). The phylogenetic analysis of the *16S rRNA* gene of *A*. *capra* revealed three distinct clusters with 99–100% homology with other isolates from different countries. Our isolates showed higher similarity with isolates from China (KM206273, KP314237, MT799937), Pakistan (ON238129, ON238130, ON238131), Angola (MT898988), India (MZ558066), Iran (MW692362), and Turkey (MT632469) isolated from human, sheep, ticks, goats, cattle, Gaddi goat, Persian Onager (*Equus hemionus onager*), and Turkish goats, respectively. In conclusion, *A*. *capra* is endemic in Punjab, Pakistan, there is a need to conduct large scale surveillance studies to assess the status of this pathogen at human-animal interface as well as to develop effective preventive and control strategies to reduce the economic losses associated with anaplasmosis in small ruminants.

## Introduction

Livestock is the major subsector of agriculture, contributing 62.68% to agriculture and 14.36% to the national gross domestic product (GDP) of Pakistan. Over 8 million village families are engaged in livestock rearing, driving 35–40 percent of their earnings from livestock [1]. Pakistan is the third largest goat producing country in the world with 84.7 million heads. Based on sheep production, Pakistan is the 12^th^ largest country with 32.3 million populations [1, 2]. In general, tick-borne diseases (TBDs) are the biggest economic threat to livestock production across the world. Different blood pathogens including *Anaplasma*, *Borrelia*, *Ehrlichia*, *Babesia*, *Theileria* and Louping ill virus are the most frequent TBDs in ruminants causing considerable economic losses [3–7].

Anaplasmosis is one of the most prevalent vector-borne illness of domestic and wild animals and humans all over the world including Pakistan [8–12]. There are seven recognized species in the genus *Anaplasma viz*. *A*. *marginale*, *A*. *centrale*, *A*. *bovis*, *A*. *ovis*, *A*. *platys*, *A*. *phagocytophilum* and *A*. *caudatum* [13]. In addition to this, the researchers also proposed additional species of *Anaplasma* including *A*. *odocoilei*, *A*. *capra* and *Anaplasma* sp. ’Omatjenne’ [14]. Of the aforementioned species of *Anaplasma*, *A*. *ovis*, *A*. *capra* and *A*. *phagocytophilum* are the major species responsible for anaplasmosis in sheep and goats [4, 15].

A new zoonotic tick-borne *Anaplasma* that gained the attention of veterinary and public health researchers provisionally named as *A*. *capra* [16]. This organism was the first identified in goats in central and northern China [17]. Years later, this was detected in human hospitalized patients of Mudanjiang Forestry Central Hospital, Heilongjiang, China. In addition, twenty-eight human cases have been reported in Heilongjiang Province in northeast China. However, this organism is not yet recognized as a separate species. *Anaplasma capra* is an emerging zoonotic pathogen and phylogenetically distinct from other *Anaplasma* species [18]. The disease caused by this organism is non-specific signs with fever, malaise, headache, rash, eschar, dizziness, and chills in humans [18, 19]. The disease caused by *Anaplasma* species exhibit some level of host specificity, but this feature is distorted due to the detection of *Anaplasma* in various hosts, which further complicates the epidemiology of the disease. *A*. *capra* can infect ruminants like sheep, goat, water buffalo [18, 20, 21]; wild animals like Muntjac (*Muntiacus muntjak*), Japanese Serow (*Capricornis crispus*), deer, Korean water deer (*Hydropotes inermis argyropus*), Roe dear (*Capreolus capreolus*), Persian onegar (*Equus hemionus onager*) [22–24]; humans [18]; dogs [25]; mouflon sheep-*Ovis gmelini* [26] and ticks [27, 28].

Although *A*. *capra* was first detected in goats (*Capra aegagrus hircus*) using *msp4* and *16S rRNA* genes in central and southern China [29] and further studies revealed the occurrence of this pathogen in Pakistan [26], France [23], Turkey [21, 30], Japan [22, 31], Korea [32], Spain [24, 33], South Korea [32, 34], Ghana [28] and Italy [35]. It has also been reported in goats from Sweden [36], Greece [37], Pakistan [38] and Kyrgyzstan [39, 40] by amplifying *groEL*, *gltA* and *16S rRNA* genes. The infected animals mostly remain asymptomatic and show non-specific signs. These common signs would be very difficult to distinguish clinically from other tick-borne illnesses.

The major ixodid ticks that biologically transmit *Anaplasma* are *Ixodes*, *Dermacentor*, *Rhipicephalus*, and *Amblyomma* which transmit the intracellular rickettsiales to their mammalian hosts [38, 41]. The organism is transmitted by tick bite, transplacentally, and blood-contaminated needles [38, 42, 43]. Additionally, the risk of mechanical transmission by the bite of flea and termite bites also exists [44]. Regardless of competent vectors, *A*. *capra* has been detected in ticks such as *Rhipicephalus microplus*, *Ixodes persulcatus*, *Dermacentor abaensis*, *D*. *nuttalli*, *Haemaphysalis longicornis* and *H*. *qinghaiensis* [45–47]. However, the competent vectors of *A*. *capra* are not known yet [30, 46].

Multiple risk factors such as environmental (area, climate, temperature, rainfall, competent vector population, reservoirs, habitat, mechanical insect vectors, season, humidity, altitude, vegetation cover); host (sex, breed, age, gestation, tick infestation, history of disease, body condition score, health status, carriers, drug resistance), and managemental (flock size, housing, floor, use of acaricide, grazing system, hygiene, animal movement, stall feeding, agricultural and animal husbandry practices, contaminated fomites) are associated with the occurrence of anaplasmosis [48–54].

Giemsa-stained blood smears are frequently used to identify *Anaplasma* infected animals. Although microscopy is affordable and still regarded as the best, yet it is a laborious technique with low accuracy and requires a skilled examiner [55]. Various diagnostic molecular techniques have been utilized for the diagnosis of anaplasmosis such as PCR [18], RFLP-PCR [56], Nested PCR [30], RLB [57] and sequencing [30]. Globally, different genes have been targeted for molecular characterization of *A*. *capra*, such as a major surface protein 4 (*msp4*) [20], *groEL* [47], *gltA* [18, 30] and *16S rRNA* [18, 26]. Pakistan’s climate, vegetation, and geographic conditions favor the existence and growth of ticks. Defining the regional status of new zoonotic genotypes of tick-borne pathogens is important at disease control standpoint. Epidemiological and risk factors information with respect to zoonotic *A*. *capra* in sheep and goats are scarce. There is only one report of *A*. *capra* in mouflon (*Ovis gmelini*) and domestic sheep (*Ovis aries*) from Pakistan which differ in terms of sample size and study area. Therefore, the present study was aimed to estimate the prevalence, associated determinants on anaplasmosis and phylogenetic investigation of *A*. *capra* in sheep and goats from Faisalabad district, Pakistan.

## Materials and methods

### Study area

**Fig 1:**
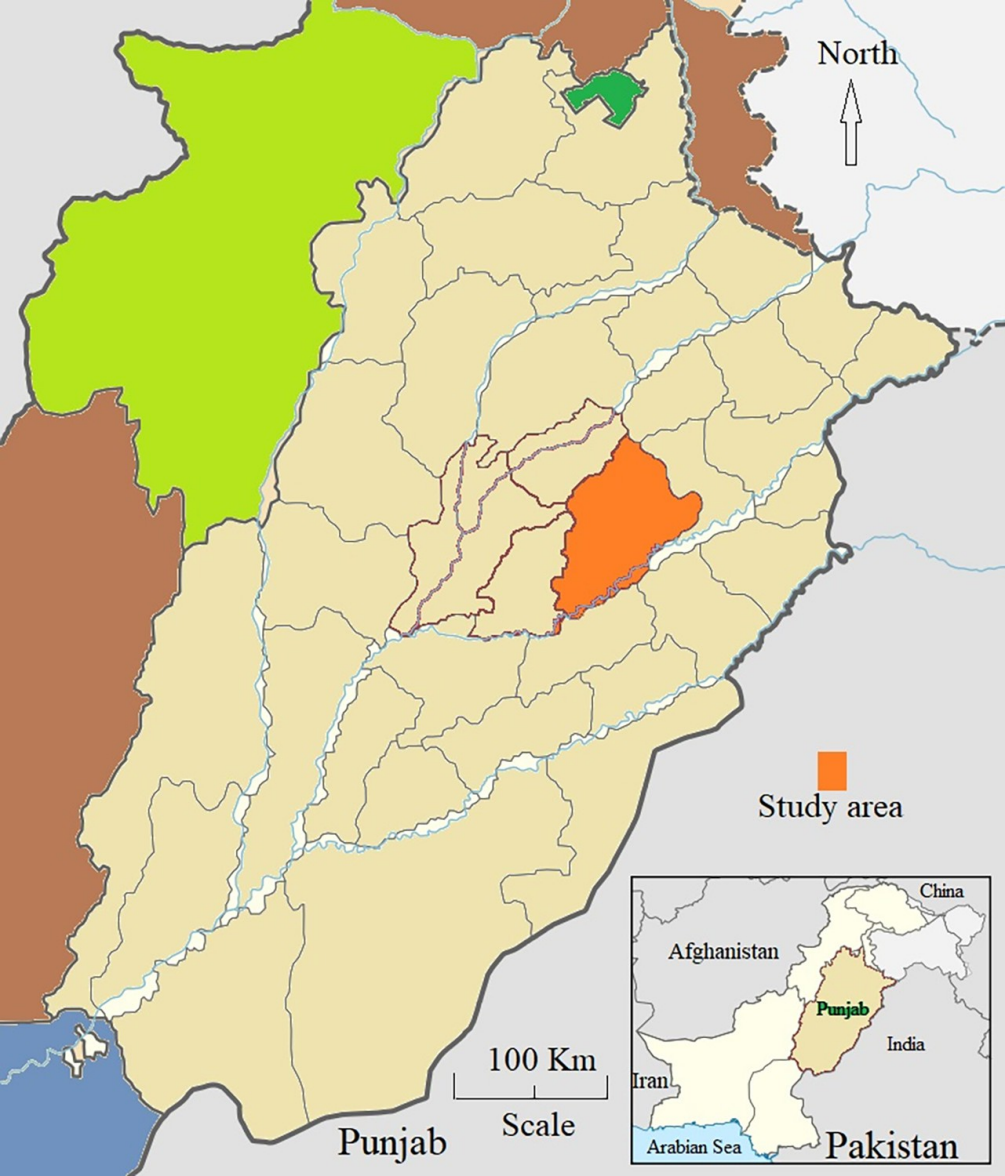
Map of the study area.

Faisalabad district is located between Ravi and Chenab Rivers in the central part of Punjab province, coordinated at 31°25′05.10″N 73°04′39.27″E, and an elevation of 184 meters above sea level (603 feet). The Faisalabad district is indicated in orange color in Fig 1. The raw map was retrieved from https://en.wikipedia.org/wiki/Faisalabad_District#/media/File:Pakistan_-_Punjab_-_Faisalabad.svg. The district has five administrative units/tehsils; namely, Saddar, Sammundari, Tandlianwali, Jaranwala, and Chak Jhumra. The Faisalabad has extended summer with warm (March, November), hot (April, October), sweltering (May to September) and comfortable (January, February, December) weather. The average annual high temperature during summer ranges from 93–104°F (33.9–40°C), while winter is short, dry, cool, and mostly clear with an average low temperature ranging from 44–46°F (6.7–7.8°C). The annual rainfall in Faisalabad is 1.05 inches (26.67 mm) (available at https://weatherspark.com).

### Study design

The current study was carried out in the Faisalabad district, Pakistan, from January 2022 to May 2022. The study was approved as per ethical guidelines of Graduate Studies and Research Board, Directorate of Graduate Studies, The University of Agriculture Faisalabad, Pakistan, vide letter no. DGS/10925-28, dated March 28, 2022. A total of 384 blood samples were collected from small ruminants (234 goats and 150 sheep) after consent from animal owners using a simple random sampling method. A sample size of 384 was calculated assuming 50% prevalence at 95% confidence using the formula as described by Thrusfield [58].

### Risk factors estimation

For estimation of epidemiological risk factors, a pretested questionnaire was filled to gather information after consent at the time of sample collection from each animal owner/farm manager regarding the biotic and abiotic risk factors of age (<2 years and >2 years), gender (male and female), species (goat and sheep), housing type (concrete and muddy), tick infestation (yes and no), hygiene at farm (poor and good), feeding pattern (animals at farm with zero grazing and free range grazing), previous tick history (yes and no), tick control practices (yes and no) and mix farming (present and absent) associated with anaplasmosis. The questionnaire having close-ended questions was completed on spot at the time of blood sampling for each animal.

### Blood smear examination

A 5–7 ml blood was drawn aseptically from the jugular vein of sheep and goats with the sterile syringe and shifted into ethylene diamine tetra acetic acid (EDTA) coated vacutainers for conventional assay. From each blood sample, a thin blood smear was prepared as described by Atif et al. [59] and the smear was examined at 100X under oil immersion lens and at least 50 fields per slide were examined to declare a sample as positive or negative [60].

### DNA extraction

One milliliter of blood was collected aseptically from EDTA coated vacutainers individually for DNA extraction. The samples were moved to Laboratory of Veterinary Preventive Medicine and Public Health, Department of Clinical Medicine and Surgery, University of Agriculture, Faisalabad under cold chain. The DNA was extracted according to the manufacturer’s instructions using the commercial QIAamp® DNA Mini Kit (Qiagen, Germany; catalog No. 69504). A total of 200 ul of individual blood samples were used to extract DNA. The extracted DNA was kept at -40°C until further testing after being measured for purity and concentration using Nano-drop at 260/280 nm for all samples.

### PCR amplification and gel electrophoresis

The PCR was performed targeting *16S rRNA* gene of *Anaplasma* using forward primer HER 16SF (5’-GGTACCYACAGAAGAAGTCC-3’), and reverse primers HER 16SR (5’-TAGCACTCATCGTTTACAGC–3’), as described by Saleem *et al*. [61]. Final reaction volume of PCR was 20 ul and each reaction contained 10ul Vazyme blue premix (catalog No. P222), A 1 μl forward primer, 1 μl reverse primer, 4 ul water, and 4 ul template DNA was used. Positive and negative controls were also used for the validation of each test. Amplicons were subjected to 2% gel electrophoresis by using 1 μl gel red solution. After solidification, the tray along with the gel was transferred to the electrophoresis tank. The 50 bp DNA ladder (Takara) was loaded in the first well and the samples were loaded into the remaining wells. A 5ul PCR amplicon was loaded in each well of the gel tank, connected to the Power Pac (Bio-Rad, USA) and set to 100–110 volts for electrophoresis at 45–60 minutes. The PCR results were observed with a UV illuminator.

### Sequencing and phylogenetic analysis

To validate the presence of *Anaplasma* specie, four representative PCR positive products were sent for sequencing. Amplified fragments were purified using Gel Extraction Kit (Thermo Scientific) and shipped to Lab Genetix, Lahore for sequencing. Previously published *16S rRNA* sequences of *A*. *capra* (KM206273, KP314237, KP314238, KU879112, LC432123, LC432126, MF066918, MH762075, MT632469, MT799937, MT898988, MW692362, MW721591, MW930537, MZ558066, OK091152, ON238129, ON238130, ON238131, OQ248254) from china, Argentina, South Korea, Turkey, Angola, Iran, France, Pakistan, India, Portugal and Ghana were retrieved from the GenBank database (https://www.ncbi.nlm.nih.gov/genbank/]. The selected sequences and isolates from the current study were aligned with MUSCLE. Bootstrap values were determined by using 500 replications. A phylogenetic tree was constructed based on Maximum Likelihood method and Tamura 3-parameter model on MEGA11 [62, 63].

### Statistical analysis

Chi square test was performed to determine the association of infection with anaplasmosis and disease determinants. Univariable analysis was used to determine the relationship between anaplasmosis and variables of age (<2 years and >2 years), gender (male and female), species (goat and sheep), housing type (Concrete and muddy), tick infestation (yes and no), hygiene at farm (poor and good), feeding pattern (farmed and free range), previous tick history (yes and no), tick control status (yes and no) and mix farming (present and absent). Multivariable analysis was performed for the variables with values *p*<0.2 to determine risk factors, odds ratio, and confidence intervals for each variable. The *p*-value less than or equal to 0.05 was considered as significant. The statistical data was analyzed with IBM SPSS Statistics 26.

## Results

### Epidemiology

All microscopic positive samples were subjected for further confirmation using PCR. The PCR based overall prevalence of anaplasmosis was 34.11% (131/384). The product of 345bp was perceived on the agarose gel using UV gel illuminator. All the microscopy based positive samples were also found positive from PCR. In the present study, anaplasmosis was more common in goats than sheep. Out of 131 positive animals, 98 goats and 33 sheep were positive for anaplasmosis. The inclusive prevalence of caprine and ovine anaplasmosis was 41.88% and 22%; respectively (Table 1). The Chi square analysis (*p*<0.05) illustrated that the species-wise prevalence of anaplasmosis was statistically significant (*X*^2^ = 59.93, df = 1, P = 0.000). All PCR positive animals were also found positive based on blood smear microscopy and demonstrated intra-erythrocytic inclusion bodies.

**Table 1:** Prevalence and determinants of *Anaplasma* in sheep and goats from Faisalabad district, Pakistan.

Factor	Category	Total	Positive	Prevalence (%)	Chi-square	*p*-value
Specie	Sheep	234	98	41.88	59.93	0.000
	Goat	150	33	22.00		
Gender	Male	110	17	15.45	100.10	0.000
	Female	274	114	41.61		
Age	<2 years	208	88	42.31	40.29	0.000
	>2 years	176	43	24.43		
Housing type	Concrete	104	25	24.04	78.61	0.000
	Muddy	280	106	37.86		
Tick infestation	Yes	292	110	37.67	88.99	0.000
	No	92	21	22.83		
Hygiene at Farm	Poor	224	93	41.52	51.59	0.000
	Good	160	38	23.75		
Feeding pattern	Zero grazing	125	35	28.00	62.12	0.000
	Grazing	259	96	37.07		
History of TBDs*	Yes	234	92	39.32	59.93	0.000
	No	150	39	26.00		
Tick control practices	Yes	234	100	42.74	64.35	0.000
	No	150	31	20.67		
Other species at farm	Present	214	69	32.24	14.70	0.000
	Absent	170	62	36.47		

*TBDs = Tick-borne Diseases

The results indicated that *Anaplasma* is more conjoint in adults than young animals. On the basis of age, animals were categorized into two groups (<2 years and >years). Prevalence was greater in <2 years of age (42.31%; 88/208) than >2 years (24.43%; 43/176) age groups. A significant association was found among different age groups (*X*^2^ = 40.29, df = 1, *p* = 0.000). Gender-wise frequency of disease had a significant association (*X*^2^ = 100.10, df = 1, *p* = 0.000) indicating a higher positivity in females (41.61%; 114/274) than males (15.45%; 17/110), regardless of host type. The animals were screened for tick infestation to assess the role of ticks on disease outcome. The animals infested with ticks had higher disease positivity rates than tick-free animals with significant association (*X*^2^ = 88.99, df = 1, *p* = 0.000). All hypothesized risk factors were statistically significant (Table 1). Furthermore, when compared to adult, young animals experienced the disease more frequently. According to univariate and multivariate analysis gender of small ruminants was a statistically substantial risk factor. The analysis of univariate (*p* = 0.02, OR = 3.532, CI = 1.201–10.387) and multivariate (*p* = 0.000, OR = 1.757, CI = 1.305–2.366) logistic regression expressed gender as the significant risk factor (Table 2).

**Table 2:** Univariate and multivariate analysis of risk factors associated with molecular prevalence of *Anaplasma* in sheep and goats from Faisalabad district, Pakistan.

**Univariate analysis**						
**Variable**		**Β** ^1^	***p*-value**	**Odds ratio**	**95% Confidence Interval (CI)**	
					**Lower CI**	**Lower CI**
Specie		1.213	0.291	3.364	0.355	31.919
Gender		1.262	0.022	3.532	1.201	10.387
Age		18.34	40.29	92052617	0.000	--
Housing type		-23.24	0.999	0.000	0.000	--
Tick infestation		20.70	0.999	974229071	0.000	--
Hygiene at farm		-23.21	0.999	0.000	0.000	--
Feeding pattern		21.46	0.997	2082630627	0.000	--
History of TBDs^2^		-38.24	0.999	0.000	0.000	--
Tick control practices		1.166	1.000	3.210	0.000	--
Other species at farm		20.06	0.997	514201222	0.000	--
**Multivariate analysis**						
**Variable**	**Category**	**Β** ^**1**^	***p*-value**	**Odds ratio**	**95% Confidence Interval (CI)**	
					**Lower CI**	**Upper CI**
Gender	Male*	--	--	--	--	--
	Female	0.564	0.000	1.757	1.305	2.366
Other species at farm	Absent*	--	--	--	--	--
	Present	-1.138	0.000	0.321	0.237	0.434

^1^Regression coefficient

^2^Tick-borne diseases

**Statistically significant

### Sequencing and phylogenetic analysis

The PCR results based on amplification of *16S rRNA* gene of Anaplasma had revealed 131 positive animals out of 384 samples. The representative sequences (n = 04) were sent for sequencing and displayed 99–100% identity with the *Anaplasma* species. The acquired sequences revealed novel isolates of *A*. *capra* with Genbank accession numbers ON834323, ON838209, ON838210, and ON838211. The phylogenetic analysis of the *16S rRNA* gene of *A*. *capra* revealed three distinct clusters with 99–100% similarity with other isolates from different countries.

**Fig 2:**
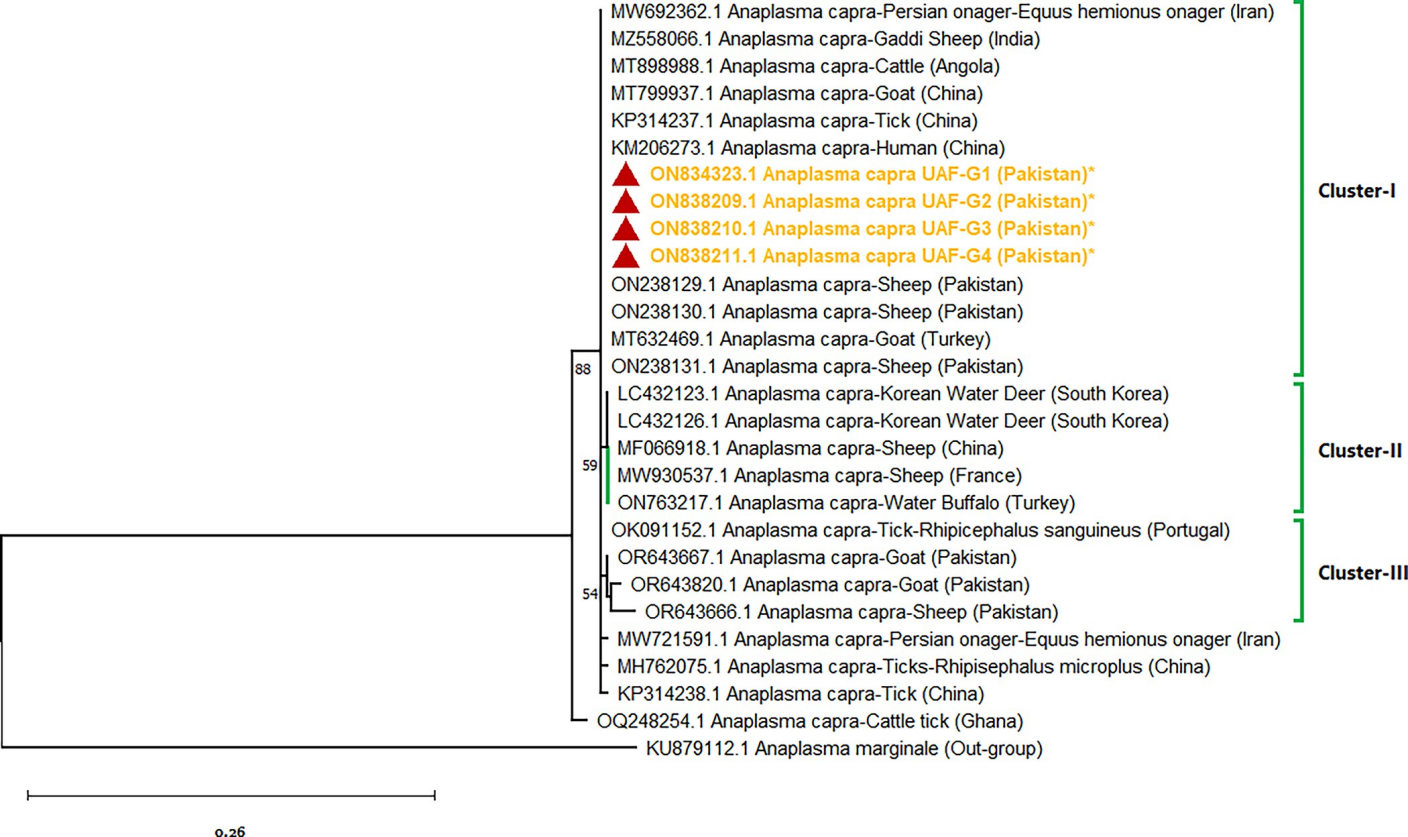
Phylogenetic tree based on 16SrRNA gene sequences (345bp) of *A*. *capra* isolated from Pakistani small ruminants in comparison to globally isolated sequences.

Our isolates are grouped in cluster-I and showed higher similarity with isolates from China (KM206273, KP314237, MT799937), Pakistan (ON238129, ON238130, ON238131), Angola (MT898988), India (MZ558066), Iran (MW692362), and Turkey (MT632469) isolated from human, sheep, ticks, goats, cattle, Gaddi goat, Persian Onager (*Equus hemionus onager*), and Turkish goats. Cluster-II consisted of Isolates of South Korea (LC432123, LC432126), China (MF066918), France (MW930537) and Turkey (ON763217) isolated from Korean Water Deer, sheep, and water buffalo, respectively. Cluster-III comprised of isolates from Portugal (OK091152), Pakistan (OR643820, OR643666, OR643667) collected from *Rhipicephalus sanguineus* ticks, goats and sheep, respectively. The isolates of current study are mentioned with red triangles and highlighted in bold with asterisks (Fig 2).

## Discussion

The primary sources of financial losses for resource-limited livestock farmers in impoverished nations are vectors and vector-borne diseases. Small ruminants are the major source of income for marginal and small farming communities that are heavily dependent on the environment. Due to global warming and climate change, ectoparasites are spreading their habitat to newer areas, putting the previously unexposed hosts at risk of various tick-transmitted pathogens. Anaplasmosis is one of the most significant vector-borne illnesses at human-animal interface. Recently, *A*. *capra* got attention from veterinary and public health researchers, and detected in Asian and European countries [16, 23, 64–66]. The *A*. *capra* is a zoonotic pathogen also detected in tick vectors such as *Haemaphysalis longicornis*, *H*. *qinghaiensis*, *Dermacentor abaensis*, *D*. *nuttalli* and *Rhipicephalus microplus* [37, 38, 41]. They have been isolated from humans and goats inhabiting forests in China [18, 33]. *Anaplasma capra* has been reported from deer, sheep, and goats in France [23, 67] and Onagers (*Equidae*) in Iran [65]. In Pakistan, only one study which depicted *A*. *capra* in domestic and Mouflon sheep (*Ovis gmelini*) which differ in terms of small sample size, outcome, and study location [26]. In the first surveillance study, we performed molecular detection, estimated risk factors, and conducted phylogenetic analysis in small ruminants from Faisalabad district, Pakistan.

According to PCR results, the overall prevalence of anaplasmosis in small ruminants was 34.11%. Likewise, earlier reports of anaplasmosis among small ruminants from Korea (10.8%) [34]; Turkey (31.4%) [46], China (30.1 to 59.7%) [68, 69], Pakistan (21.7%) [48] and China (29.1%) [70] were in line with our findings. Additionally, *A*. *capra* was found in 10 out of 452 (2.2%) samples from goats in South Korea [34], 79 of 435 (18.2%) samples from sheep in China [68], 13 of 224 (5.8%) from cattle in Malaysia [71], 5 of 155 (3.22%) from sheep, and 0 of 72 from goats in Turkey. Likewise, 40 goats and 46 horses in Malaysian research were found free from *A*. *capra* [71]. However, lower prevalence was depicted from Korea, Spain and Turkey ranging from 0.5 to 5.8 percent detected from goats and Cervids [24, 72–74].

The results of the present investigation were similar to Belkahia et al. [75], where they depicted higher disease occurrence in female than male animals. This determinant was proved as the most significant risk factor based on univariate analysis. These findings were also supported by Atif et al. [12] as they mentioned higher infection in females than males. This result can be justified by the fact that immunosuppression in females during pregnancy or lactation could be responsible for higher disease outcome in female animals. Belkahia et al. [75], who mentioned a higher infection rates in ewes than in rams. Furthermore, it was revealed that young animals were more infected than adults. Age-related findings are incoherent with Atif et al. [12], who indicated that adults had higher infectivity than young animals. Similarly, the occurrence of anaplasmosis was significantly higher in sheep than goats. As anticipated, anaplasmosis risk was greater in tick-infected animals in this research than non-tick infested animals. Rahman et al. [76], supported our findings who reported comparable results in Jamnapari breed of goats from Bangladesh. Additionally, animals having a history of tick illness had higher disease positivity rates than those who had no history of disease. Our findings are corroborated by the research of Farooqi et al. [77], conducted on cattle in Northern, Pakistan. The mixed farming yield higher infectivity for *Anaplasma* infection. This had been the fact that animals housed in the same facility might get tick-borne diseases from various animal species.

The highest prevalence was observed in free-grazing animals (39.6%), followed by stall-fed animals (36.5%), and the lowest infectivity in semi-grazing animals (23.9%). Chi square test specified a significant linking between grazing pattern and disease outcome in small ruminants, while regression analysis revealed a non-significant effect. In addition, Yan and colleagues depicted that animals are more prone to infection and the occurrence of anaplasmosis was associated with free-grazing [43]. The grazing could have augmented the contact between vectors and other animals [78]. Previous studies conducted in Pakistan suggested that grazing animals were at higher risk of infection than their non-grazing counterparts [49].

The acquired sequences revealed four novel isolates of *A*. *capra*. The phylogenetic analysis of the *16S rRNA* gene of *A*. *capra* indicated three distinct clusters with 99–100% similarity with other isolates from different countries. Our isolates showed higher homology with isolates from China (KM206273, KP314237, MT799937), Angola (MT898988), Iran (MW692362.1), India (MZ558066) and Portugal (OK091152.1) isolated from human, tick, goat, cattle, Persian onager (*Equus hemionus onager*), Gaddi goat and *Rhipicephalus microplus* ticks, respectively. This is the first report of *A*. *capra* in goats from Pakistan. To date, the pathogens remained unnoticed and require large scale surveillance of migratory and non-migratory small ruminants to estimate the gravity of the problem with special reference to vector-transmitted hemoparasites.

Current surveillance studies concluded that zoonotic *A*. *capra* is prevalent in small ruminants in Faisalabad district, Pakistan. Gender is the major risk factor for *A*. *capra* infection. Phylogenetic analysis indicated novel genotypes. Further studies should be conducted on genetic diversity, host specificity, pathogenicity, vectors competence, and transmission attributes of *A*. *capra* zoonotic anaplasmosis using specie specific primers (such as *gltA* and *groEL* genes) [79, 80] for effective prevention and control of emerging zoonotic pathogen.

## Supporting information

S1 Data (RAR)

S2 Data (RAR)

## References

[1] Economic Survey of Pakistan, Government of Pakistan, Finance Division, Economic Advisor Wing, Islamabad. Chapter 2/Agriculture, 2023, p. 27. Available at: https://www.finance.gov.pk/survey/chapters_23/02_Agriculture.pdf. Accessed 24 September, 2023.

[2] Khan M, Khan M, Ahmad S, Mahmood S. Genetic resources and diversity in Pakistani sheep. Int J Agric Biol. 2007; 9:941–944. https://www.researchgate.net/publication/237318075_Genetic_Resources_and_Diversity_in_Pakistani_Goats.

[3] Berggoetz M, Schmid M, Ston D, Smith V, Chevillon C, Pretorius AM. Tick-borne pathogens in the blood of wild and domestic ungulates in South Africa, Interplay of game and livestock. Ticks Tick Borne Dis. 2014; 5:166–75. doi: 10.1016/j.ttbdis.2013.10.007 . 24418761

[4] Khan A, Muhammed AA, Nasreen N, Iqbal F, Cossio-Bayugar R, Ali Sha SS, et al. Tick-borne haemoparasitic diseases in small ruminants in Pakistan: Current knowledge and future perspectives. Saudi J Biol Sci. 2022; 29:2014–2025. doi: 10.1016/j.sjbs.2021.12.046 . 35531246 PMC9072882

[5] Khan A, Nasreen N, Niaz S, Sajjad Ali Shah S, Mitchell III-RD, Ayaz S, et al. Tick burden and tick species prevalence in small ruminants of different agencies of the Federally Administered Tribal Areas (FATA), Pakistan. Intl J Acarol. 2019; 45:374–80. 10.1080/01647954.2019.1663930.

[6] Lihou K, Rose Vineer H, Wall R. Distribution and prevalence of ticks and tick-borne disease on sheep and cattle farms in Great Britain. Parasit Vectors. 2020; 13:406. doi: 10.1186/s13071-020-04287-9 . 32778148 PMC7419194

[7] Basit MA, Ijaz M, Abbas RZ, Khan JA, Ashraf K. First Molecular evidence of Ehrlichia Infection: An emerging pathogen of small ruminants in Pakistan. Pak Vet J. 2022; 42:208–214. 10.29261/pakvetj/2022.012.

[8] Ceylan O, Uslu A, Oztürk O, Sevinc F. Serological investigation of some vector-borne parasitic and rickettsial agents in dogs in the western part of Turkey. Pak Vet J. 2021; 41:386–392. 10.29261/pakvetj/2021.052.

[9] Ceylan C, Ekici ÖD. Molecular investigation of ovine and caprine anaplasmosis in south-Eastern Anatolia region of Turkey. Pak Vet J. 2022; 43:139–145. 10.29261/pakvetj/2022.070.

[10] Abbas SN, Ijaz M, Abbas RZ, Saleem MH, Mahmood AK. Molecular characterization, risk factor analysis and hematological alterations associated with *Anaplasma phagocytophilum* in domestic cats of Pakistan. Pak Vet J. 2023; 43:493–499. 10.29261/pakvetj/2023.082.

[11] Atif FA, Abbas RZ, Mehnaz S, Qamar MF, Hussain K, Nazir MU, et al. First report on molecular surveillance based on duplex detection of *Anaplasma marginale* and *Theileria annulata* in dairy cattle from Punjab, Pakistan. Trop Anim Health Prod. 2022; 54:155. doi: 10.1007/s11250-022-03158-y . 35362760

[12] Atif FA, Ullah S, Cossío-Bayúgar R, Kashif M, Khan AU, Wu WF. Molecular epidemiology, seasonality and phylogenetic investigations of *Anaplasma ovis* in small ruminants from diverse agro-climatic regions of Punjab, Pakistan. Microorganisms 2023; 11:2430. doi: 10.3390/microorganisms11102430 . 37894088 PMC10608874

[13] Dumler JS, Barbet AF, Bekker CP, Dasch GA, Palmer GH, Ray SC, et al. Reorganization of genera in the families *Rickettsiaceae* and *Anaplasmataceae* in the order Rickettsiales: unification of some species of *Ehrlichia* with *Anaplasma*, Cowdria with *Ehrlichia* and *Ehrlichia* with Neorickettsia, descriptions of six new species combinations and designation of Ehrlichia *equi* and ’HGE agent’as subjective synonyms of *Ehrlichia phagocytophila*. Int J Syst Evol. 2001; 51:2145–2165. doi: 10.1099/00207713-51-6-2145 . 11760958

[14] Calchi AC, Vultão JG, Alves MH, Yogui DR, Desbiez AL, De Santi M, et al. Ehrlichia spp. and *Anaplasma* spp. in Xenarthra mammals from Brazil, with evidence of novel ‘Candidatus Anaplasma spp.’. Sci Rep. 2020; 10:12615. doi: 10.1038/s41598-020-69263-w . 32724088 PMC7387473

[15] Dumler Gofton AW, Doggett S, Ratchford A, Oskam CL, Paparini A, Ryan U, et al. Bacterial profiling reveals novel “Ca. Neoehrlichia, Ehrlichia”, and *Anaplasma* species in Australian human-biting ticks. PloS One 2015; 10:145–449. doi: 10.1371/journal.pone.0145449 . 26709826 PMC4692421

[16] Peng Y, Lu C, Yan Y, Shi K, Chen Q, Zhao C, et al. The first detection of *Anaplasma capra*, an emerging zoonotic *Anaplasma* sp., in erythrocytes. Emerg Microbes Infect. 2021; 10:226–34. doi: 10.1080/22221751.2021.1876532 . 33446064 PMC7894429

[17] Liu Z, Ma M, Wang Z, Wang J, Peng Y, Li Y, et al. Molecular survey and genetic identification of *Anaplasma* species in goats from central and southern China. Appl Environ Microbiol. 2012; 78: 464–470. doi: 10.1128/AEM.06848-11 . 22057867 PMC3255723

[18] Li H, Zheng YC, Ma L, Jia N, Jiang BG, Jiang RR, et al. Human infection with a novel tick-borne *Anaplasma* species in China: a surveillance study. Lancet. Infect. Dis. 2015; 15:663–670. doi: 10.1016/S1473-3099(15)70051-4 . 25833289

[19] Dahlgren FS, Heitman KN, Behravesh CB. Undetermined human ehrlichiosis and anaplasmosis in the United States, 2008–2012: A catch-all for passive surveillance. Am J Trop Med Hyg. 2016; 94:299. doi: 10.4269/ajtmh.15-0691 . 26621564 PMC4751933

[20] Yang J, Liu Z, Niu Q, Liu J, Han R, Guan G, et al. A novel zoonotic Anaplasma species is prevalent in small ruminants: potential public health implications. Parasit. Vectors 2017; 10:1–6. doi: 10.1186/s13071-017-2182-9 . 28558749 PMC5450374

[21] Sahin OF, Erol U, Altay K. Buffaloes as new hosts for *Anaplasma capra*: molecular prevalence and phylogeny based on gtlA, groEL, and 16S rRNA genes. Res Vet Sci. 2022; 152:458–464. doi: 10.1016/j.rvsc.2022.09.008 . 36148715

[22] Sato M, Nishizawa I, Fujihara M, Nishimura T, Matsubara K, Harasawa R. Phylogenetic analysis of the 16S rRNA gene of *Anaplasma* species detected from Japanese serows (*Capricornis crispus*). J Vet Med Sci. 2009; 71:1677–1679. doi: 10.1292/jvms.001677 . 20046041

[23] Jouglin M, Blanc B, De La Cotte N, Bastian S, Ortiz K, Malandrin L. First detection and molecular identification of the zoonotic *Anaplasma capra* in deer in France. PLoS One 2019; 14:e0219184. doi: 10.1371/journal.pone.0219184 . 31276519 PMC6611577

[24] Remesar S, Prieto A, García‐Dios D, López‐Lorenzo G, Martínez‐Calabuig N, Díaz‐Cao JM, et al. Diversity of *Anaplasma* species and importance of mixed infections in roe deer from Spain. Transbound Emerg Dis. 2022; 69: e374–e385. doi: 10.1111/tbed.14319 . 34529897

[25] Shi K, Li J, Yan Y, Chen Q, Wang K, Zhou Y, et al. Dogs as new hosts for the emerging zoonotic pathogen *Anaplasma capra* in China. Front Cell Infect Microbiol. 2019; 9:394. doi: 10.3389/fcimb.2019.00394 . 31850236 PMC6901931

[26] Ishaq M, Ijaz M, Lateef M, Ahmed A, Muzammil I, Javed MU, et al. Molecular characterization of *Anaplasma capra* infecting captive mouflon (*Ovis gmelini*) and domestic sheep (*Ovis aries*) of Pakistan. Small Rumin Res. 2022; 216, 106837. 10.1016/j.smallrumres.2022.106837.

[27] Guo WP, Zhang B, Wang YH, Xu G, Wang X, Ni X, et al. Molecular identification and characterization of *Anaplasma capra* and *Anaplasma platys*-like in *Rhipicephalus microplus* in Ankang, Northwest China. BMC Infect Dis. 2019; 19:1–9. doi: 10.1186/s12879-019-4075-3 . 31101084 PMC6525361

[28] Addo SO, Baako BOA, Bentil RE, Addae CA, Behene E, Asoala V, et al. Molecular survey of Anaplasma and Ehrlichia species in livestock ticks from Kassena-Nankana, Ghana; with a first report of *Anaplasma capra* and *Ehrlichia minasensis*. Arch Microbiol. 2023; 205:92. doi: 10.1007/s00203-023-03430-1 . 36795247

[29] Peng Y, Wang K, Zhao S, Yan Y, Wang H, Jing J, et al. Detection and phylogenetic characterization of *Anaplasma capra*: an emerging pathogen in sheep and goats in China. Front Cell Infect Microbiol. 2018; 8:283. doi: 10.3389/fcimb.2018.00283 . 30214896 PMC6126426

[30] Altay K, Erol U, Sahin O.F. The first molecular detection of *Anaplasma capra* in domestic ruminants in the central part of Turkey, with genetic diversity and genotyping of *Anaplasma capra*. Trop Anim Health Prod. 2022; 54:129. doi: 10.1007/s11250-022-03125-7 . 35257219

[31] Inokuma H, Brouqui P, Drancourt M, Raoult D. Citrate synthase gene sequence: a new tool for phylogenetic analysis and identification of Ehrlichia. J Clin Microbiol. 2001; 39:3031–3039. doi: 10.1128/JCM.39.9.3031-3039.2001 . 11526124 PMC88292

[32] Amer S, Kim S, Yun Y, Na KJ. Novel variants of the newly emerged *Anaplasma capra* from Korean water deer (*Hydropotes inermis argyropus*) in South Korea. Parasit. Vectors. 2019; 12:1–9. doi: 10.1186/s13071-019-3622-5 . 31345253 PMC6659236

[33] Sun XF, Zhao L, Wen HL, Luo LM, Yu XJ. Anaplasma species in China. Lancet Infect Dis. 2015; 15:1263–1264. doi: 10.1016/S1473-3099(15)00377-1 . 26531037

[34] Seo HJ, Jin BC, Kim KH, Yoo MS, Seong KW, Jeong SJ, et al. Molecular detection and phylogenetic analysis of *Anaplasma* spp. in Korean Native goats from Ulsan Metropolitan city. Vector Borne Zoonotic Dis. 2019; 19:773–776. doi: 10.1089/vbz.2018.2374 . 31355707

[35] Rocchigiani G, Ebani VV, Nardoni S, Bertelloni F, Bascherini A, Leoni A, et al. Molecular survey on the occurrence of arthropod-borne pathogens in wild brown hares (*Lepus europaeus*) from Central Italy. Infect Genet Evol. 2018; 59:142–147. doi: 10.1016/j.meegid.2018.02.005 . 29421225

[36] Lysholm S, Ådén F, Aspán A, Högberg A, Wensman JJ, Omazic A. Presence of *Anaplasma* spp. and their associated antibodies in the Swedish goat population. Animals. 2023; 13:333. doi: 10.3390/ani13030333 . 36766222 PMC9913567

[37] de Vries MA. Ticks and Tick-borne diseases surveillance with special reference to *Anaplasma* infections in small ruminants on the island of Lesvos, Greece. Research Project Veterinary Medicine Myrthe de Vries 3833828. 2018. Available from: https://studenttheses.uu.nl/handle/20.500.12932/31293.

[38] Atif FA. Alpha proteobacteria of genus Anaplasma (Rickettsiales: Anaplasmataceae): Epidemiology and characteristics of *Anaplasma* species related to veterinary and public health importance. Parasitol. 2016; 143:659–685. doi: 10.1017/S0031182016000238 . 26932580

[39] Altay K, Erol U, Sahin OF, Aytmirzakizi A. First molecular detection of *Anaplasma* species in cattle from Kyrgyzstan; molecular identification of human pathogenic novel genotype *Anaplasma capra* and *Anaplasma phagocytophilum* related strain. Ticks Tick Borne Dis 2022; 13:101861. doi: 10.1016/j.ttbdis.2021.101861 . 34773849

[40] Altay K, Erol U, Sahin OF, Aytmirzakizi A, Temizel EM, Aydin MF et al. The detection and phylogenetic analysis of *Anaplasma phagocytophilum*-like 1, *A*. *ovis* and *A*. *capra* in sheep: *A*. *capra* divides into two genogroups. Vet Res Commun 2022; 46:1271–1279. doi: 10.1007/s11259-022-09998-1 . 36167934

[41] Younas M, Ashraf K, Rashid MI, Ijaz M, Suleman M, Chohan TA, et al. Expression and purification of recombinant multi-epitope protein of *Rhipicephalus microplus* tick and its antigenicity in rabbits. Pak Vet J. 2023. 10.29261/pakvetj/2023.086.

[42] Hosseini-Vasoukolaei N, Oshaghi MA, Shayan P, Vatandoost H, Babamahmoudi F, Yaghoobi-Ershadi MR, et al. *Anaplasma* infection in ticks, livestock and human in Ghaemshahr, Mazandaran Province, Iran. J. Arthropod Borne Dis. 2014; 8:204–211. https://jad.tums.ac.ir/index.php/jad/article/view/210 . 26114134 PMC4478432

[43] Yang J, Li Y, Liu Z, Liu J, Niu Q, Ren Q, et al. Molecular detection and characterization of Anaplasma spp. in sheep and cattle from Xinjiang, northwest China. Parasit Vectors. 2015; 8:108. doi: 10.1186/s13071-015-0727-3 . 25889906 PMC4344993

[44] Kocan KM, De La Fuente J, Blouin EF, Garcia-Garcia JC. *Anaplasma marginale* (Rickettsiales: *Anaplasmataceae*): recent advances in defining host–pathogen adaptations of a tick-borne rickettsia. Parasitol. 2004; 129:285–300. doi: 10.1017/s0031182003004700 . 15938516

[45] Qin XR, Han FJ, Luo LM, Zhao FM, Han HJ, Zhang ZT, et al. *Anaplasma* species detected in *Haemaphysalis longicornis* tick from China. Ticks Tick Borne Dis. 2018; 9:840–843. doi: 10.1016/j.ttbdis.2018.03.014 . 29567147

[46] Han R, Yang JF, Mukhtar MU, Chen Z, Niu QL, Lin YQ, et al. Molecular detection of *Anaplasma* infections in ixodid ticks from the Qinghai-Tibet Plateau. Infect Dis Poverty. 2019; 8:83–90. doi: 10.1186/s40249-019-0522-z . 30728069 PMC6366118

[47] Yang J, Liu Z, Niu Q, Liu J, Han R, Liu G, et al. Molecular survey and characterization of a novel Anaplasma species closely related to *Anaplasma capra* in ticks, northwestern China. Parasit Vectors. 2016; 9:1–5. doi: 10.1186/s13071-016-1886-6 . 27884197 PMC5123347

[48] Renneker S, Abdo J, Salih DEA, Karagenç T, Bilgiç H, Torina A, et al. Can *Anaplasma ovis* in small ruminants be neglected any longer?. Transbound Emerg Dis. 2013; 60:105–112. doi: 10.1111/tbed.12149 . 24589109

[49] Niaz S, Rahman ZU, Ali I, Cossío-Bayúgar R, Amaro-Estrada I, Alanazi AD, et al. Molecular prevalence, characterization and associated risk factors of *Anaplasma* spp. and Theileria spp. in small ruminants in Northern Pakistan. Parasit. 2021; 28. doi: 10.1051/parasite/2020075 . 33416491 PMC7792498

[50] Selim A, Attia KA, Alsubki RA, Albohairy F, Kimiko I, Said MB. The first study on the seroprevalence of *Anaplasma* spp. in small ruminants and assessment of associated risk factors in North Egypt. Vet World. 2022; 15:1221. doi: 10.14202/vetworld.2022.1221-1227 . 35765471 PMC9210854

[51] Naeem M, Amaro-Estrada I, Taqadus A, Swelum AA, Alqhtani AH, Asif M, et al. Molecular prevalence and associated risk factors of *Anaplasma ovis* in Pakistani sheep. Front Vet Sci. 2023; 10:1096418. doi: 10.3389/fvets.2023.1096418 . 37065244 PMC10095557

[52] Noaman V. Epidemiological study on *Anaplasma phagocytophilum* in cattle: molecular prevalence and risk factors assessment in different ecological zones in Iran. Preventive Vet Med. 2020; 183: 105118. doi: 10.1016/j.prevetmed.2020.105118 . 32891899

[53] Noaman V. Factors associated with *Anaplasma phagocytophilum* infection in sheep in Iran. Small Rumin Res. 2022; 208:106617. 10.1016/j.smallrumres.2022.106617.

[54] Noaman V, Sazmand A. *Anaplasma ovis* infection in sheep from Iran: molecular prevalence, associated risk factors, and spatial clustering. Trop Anim Health Prod. 2022; 54: 6. doi: 10.1007/s11250-021-03007-4 . 34890017

[55] Silaghi C, Santos AS, Gomes J, Christova I, Matei IA, Walder G, et al. Guidelines for the direct detection of *Anaplasma* spp. in diagnosis and epidemiological studies. Vector Borne Zoonotic Dis. 2017; 17:12–22. doi: 10.1089/vbz.2016.1960 . 28055579

[56] Noaman V, Shayan P. Comparison of microscopy and PCR-RFLP for detection of *Anaplasma marginale* in carrier cattle. Iranian J. Microbiol, 2010; 2:89. . 22347555 PMC3279773

[57] Kolo AO, Sibeko-Matjila KP, Maina AN, Richards AL, Knobel DL, Matjila PT. Molecular detection of zoonotic rickettsiae and *Anaplasma* spp. in domestic dogs and their ectoparasites in Bushbuckridge, South Africa. Vector-Borne Zoonotic Dis. 2016; 16:245–252. doi: 10.1089/vbz.2015.1849 . 26974185

[58] Thrusfield M. *Veterinary epidemiology*, John Wiley & Sons; Hoboken, NJ, USA; 2018.

[59] Atif FA, Khan MS, Iqbal HJ, Roheen T. Prevalence of tick-borne diseases in Punjab (Pakistan) and hematological profile of *Anaplasma marginale* infection in indigenous and crossbred cattle. Pak J Sci. 2012; 64:11–15. https://pjosr.com/index.php/pjs/article/download/467/350.

[60] Adam KMG, Paul J, Zaman V. Medical and Veterinary Protozology Churchill living stone Edinburgh and London 1971. pp. 200.

[61] Saleem S; Ijaz M; Farooqi SH; Rashid MI; Khan A; Masud A, et al. First molecular evidence of equine granulocytic anaplasmosis in Pakistan. Acta Tropica. 2018; 180:18–25. https://pubmed.ncbi.nlm.nih.gov/29306724/ doi: 10.1016/j.actatropica.2017.12.032 29306724

[62] Tamura K. Estimation of the number of nucleotide substitutions when there are strong transition-transversion and G+C-content biases. Mol. Biol. Evol. 1992; 9:678–687. doi: 10.1093/oxfordjournals.molbev.a040752 . 1630306

[63] Tamura K, Stecher G, Kumar S. MEGA11: Molecular evolutionary genetics analysis, Version 11. Mol. Biol. Evol. 2021; 38:3022–3027. doi: 10.1093/molbev/msab120 33892491 PMC8233496

[64] Wei W, Li J, Wang YW, Jiang BG, Liu HB, Wei R, et al. *Anaplasma platys*-Like infection in goats, Beijing, China. Vector Borne Zoonotic Dis. 2020; 20:755–762. doi: 10.1089/vbz.2019.2597 . 32679008

[65] Staji H, Yousefi M, Hamedani MA, Tamai IA, Khaligh SG. Genetic characterization and phylogenetic of *Anaplasma capra* in Persian onagers (*Equus hemionus onager*). Vet. Microbiol. 2021; 261:109199. doi: 10.1016/j.vetmic.2021.109199 . 34385006

[66] Remesar S, Diaz P, Prieto A, García‐Dios D, Panadero R, Fernández G, et al. Molecular detection and identification of piroplasms (Babesia spp. and Theileria spp.) and *Anaplasma phagocytophilum* in questing ticks from northwest Spain. Med. Vet. Entomol. 2021; 35:51–58. doi: 10.1111/mve.12468 . 32757238

[67] Jouglin M, Rispe C, Grech-Angelini S, Gallois M, Malandrin L. *Anaplasma capra* in sheep and goats on Corsica Island, France: A European lineage within *A*. *capra* clade II?. Ticks Tick Borne Dis. 2022; 13:101934. doi: 10.1016/j.ttbdis.2022.101934 . 35263704

[68] Ma M, Liu Z, Sun M, Yang J, Guan G, Li Y, et al. Development and evaluation of a loop-mediated isothermal amplification method for rapid detection of *Anaplasma ovis*. J Clin Microbiol. 2011; 49: 2143–2146. doi: 10.1128/JCM.02536-10 . 21471346 PMC3122716

[69] Ait Lbacha H, Alali S, Zouagui Z, El Mamoun L, Rhalem A, Petit E, et al. High prevalence of Anaplasma spp. in small ruminants in Morocco. Transbound Emerg Dis. 2017; 64:250–263. doi: 10.1111/tbed.12366 . 25916245

[70] Yang J, Han R, Niu Q, Liu Z, Guan G, Liu G, et al. Occurrence of four Anaplasma species with veterinary and public health significance in sheep, northwestern China. Ticks Tick Borne Dis. 2018; 9:82–85. doi: 10.1016/j.ttbdis.2017.10.005 . 29037826

[71] Koh FX, Panchadcharam C, Sitam FT, Tay ST. Molecular investigation of *Anaplasma* spp. in domestic and wildlife animals in Peninsular Malaysia. Vet Parasitol: Reg Stud Reports. 2018; 13:141–147. doi: 10.1016/j.vprsr.2018.05.006 . 31014863

[72] Oguz B, Deger MS, Al-Olayan E, El-Ashram S. Molecular Survey of *Anaplasma capra* in Goats in Van Province, Eastern Türkiye. Acta Parasitol. 2023; 19:1–5. doi: 10.1007/s11686-023-00758-y . 38112913

[73] Seo MG, Ouh IO, Lee H, Geraldino PJ, Rhee MH, Kwon OD, et al. Differential identification of Anaplasma in cattle and potential of cattle to serve as reservoirs of *Anaplasma capra*, an emerging tick-borne zoonotic pathogen. Vet Microbiol. 2018; 226:15–22. doi: 10.1016/j.vetmic.2018.10.008 . 30389039

[74] Miranda EA, Han SW, Cho YK, Choi KS, Chae JS. Co-infection with *Anaplasma* species and novel genetic variants detected in cattle and goats in the Republic of Korea. Pathogens. 2021; 10:28. doi: 10.3390/pathogens10010028 . 33401478 PMC7830860

[75] Belkahia H, Said MB, El Hamdi S, Yahiaoui M, Gharbi M, Daaloul-Jedidi M, et al. First molecular identification and genetic characterization of *Anaplasma ovis* in sheep from Tunisia. Small Rumin. Res. 2014; 121: 404–410. 10.1016/j.smallrumres.2014.07.009.

[76] Rahman M, Faruque MR, Rahman MM, Chowdhury MYE. Epidemiology and molecular detection of Anaplasma spp. in goats from Chattogram district, Bangladesh. Vet Med Sci. 2022; 8: 1240–1249. doi: 10.1002/vms3.775 . 35218684 PMC9122420

[77] Farooqi SH, Ijaz M, Rashid MI, Nabi H, Islam S, Aqib AI, et al. Molecular epidemiology of bovine anaplasmosis in Khyber Pakhtunkhwa, Pakistan. Trop Anim Health Prod. 2018; 50:1591–1598. https://pubmed.ncbi.nlm.nih.gov/29740781/ doi: 10.1007/s11250-018-1599-2 29740781

[78] Eisawi NM, El Hussein ARM, Hassan DA, Musa AB, Hussien MO, Enan KA, et al. A molecular prevalence survey on Anaplasma infection among domestic ruminants in Khartoum State, Sudan. Trop Anim Health Prod. 2020; 52:1845–1852. doi: 10.1007/s11250-019-02176-7 . 31938957

[79] Lin ZT, Ye RZ, Liu JY, Wang XY, Zhu WJ, Li YY, et al. Epidemiological and phylogenetic characteristics of emerging *Anaplasma capra*: A systematic review with modeling analysis. Infect Genet Evol. 2023; 115:105510. doi: 10.1016/j.meegid.2023.105510 . 37778674

[80] Altay K, Erol U, Sahin O.F. *Anaplasma capra*: a new emerging tick-borne zoonotic pathogen. Vet Res Commun, 2024; 10.1007/s11259-024-10337-9. Advance online publication. doi: 10.1007/s11259-024-10337-9 . 38424380 PMC11147849

