# Correction: Molecular surveillance based on anaplasmosis in domestic small ruminants: First report on zoonotic *Anaplasma capra* and phylogenetic insights from Faisalabad, Pakistan

**DOI:** 10.1371/journal.pone.0316866

**Published:** 2024-12-30

**Authors:** Muhammad A. Razzaq, Muhammad Imran, Farhan Ahmad Atif, Rao Z. Abbas, Mughees A. Alvi, Ayman A. Swelum, Zia-ud-Din Sindhu, Muhammad K. Khan, Muhammad A. Sabir Mughal, Adil Khan, Wen-Feng Wu

In the Funding section, the project number from the funder King Saud University’s is listed incorrectly. The correct project number is: RSPD2024R971.
